# Beneficial effects of novel antagonists of GHRH in different models of Alzheimer's disease

**DOI:** 10.18632/aging.100504

**Published:** 2012-11-29

**Authors:** Miklos Jaszberenyi, Ferenc G. Rick, Luca Szalontay, Norman L. Block, Marta Zarandi, Ren-Zhi Cai, Andrew V. Schally

**Affiliations:** ^1^ Endocrine, Polypeptide, and Cancer Institute, Miami Veterans Affairs Medical Center and South Florida VA Foundation for Research and Education, Miami, FL 33125, USA; ^2^ Department of Pathology, University of Miami, Miller School of Medicine, Miami, FL 33136, USA; ^3^ Division of Hematology/Oncology, Department of Medicine, University of Miami, Miller School of Medicine, Miami, FL 33136, USA; ^4^ Division of Endocrinology, Department of Medicine, University of Miami, Miller School of Medicine, Miami, FL 33136, USA; ^5^ On leave of absence from the Department of Pathophysiology, University of Szeged Medical School, Szeged, H-6701, Hungary

**Keywords:** Alzheimer's disease, growth hormone-releasing hormone, GHRH, 5XFAD mice, transgenic model, HCN-2 cell line, Morris water maze

## Abstract

Alzheimer's disease is the most frequent debilitating disorder of the central nervous system. Neuroendocrine mechanisms appear to play an important role in this insidiously developing disease. In the present study, the effects of a recently developed growth hormone-releasing hormone (GHRH) antagonist (MIA-690) were evaluated *in vivo* observing the behavior of genetically modified “Alzheimer's” 5XFAD mice in a Morris water maze (MWM). The effects of the antagonist were also evaluated *in vitro* using HCN2 human cortical cell cultures treated with amyloid-β_1-42_. *In vivo*, the indices of cognitive performance (latency, cumulative index etc.) were followed up for 6 months. *In vitro*, the formation of reactive oxygen species, markers of inflammatory and neurohormonal signaling were measured by fluorescent detection, PCR, and ELISA. Accumulation of amyloid-β_1-42_ rafts and τ filaments in necropsied brain samples was verified with the help of ELISA. In the MWM experiments, MIA-690 decreased escape latency, and, in the brain samples, it inhibited the concentration of amyloid-β_1-42_ and τ filaments. In cell cultures, the GHRH analog showed anti-oxidative and neuro-protective properties and inhibited the GHRH-growth hormone-insulin like growth factor axis. Our data strongly suggest the merit of further studies with GHRH analogs in models of Alzheimer's disease and in elementary clinical trials.

## INTRODUCTION

Several etiological factors have been implicated in the pathogenesis of Alzheimer's disease. These factors, in this most frequent form of dementia, lead to the activation of a cascade process that brings about neuronal death and serious decline in cognitive function. These bed-ridden patients ultimately succumb to death due to inter-current infections related to aspiration, decubitus and stagnation of urine [1].

This process involves, biochemically, a pathological cleavage of amyloid precursor protein (APP). APP, in normal circumstances, is cleft by α- and γ-secretases and takes part in axonal transport, synapse formation and synaptic repair in the CNS [2]. The abnormal, sequential processing by the beta-site amyloid precursor protein–cleaving enzymes (BACE) and γ-secretase results in amyloid-β, which is highly neurotoxic. Molecules of amyloid-β, especially the amyloid-β_1-42_ type, are prone to aggregation and accumulation in the cell membrane forming insoluble aggregates called “rafts”. Subsequently, these impair membrane conductivity, Ca^2+^ fluxes, control of the formation of reactive oxygen species (ROS), τ-protein assembly, axonal transport and the polarity of mitochondrial membrane. Ultimately, the pathologic cascade leads to Ca^2+^ toxicity, activation of apoptotic processes, inflammation and neuronal death [1]. This vicious cycle can be initiated by a wide array of triggering mechanisms that can be traced back to genetic or environmental factors [3]. Monogenic forms represent the infrequent, presenile or early-onset familial Alzheimer's disease (FAD), which is usually characterized by autosomal dominant point mutations of the genes of APP or the presenilin sub-domains of γ-secretase. Both types of mutations facilitate the accumulation of toxic amyloid-β_1-42_, due to abnormal processing or breakdown [3]. Studying FAD, in the past few years, has provided us with an indispensable means to elucidate the underlying pathological phenomena. The abundant, senile or late-onset type of Alzheimer's disease is epidemiologically sporadic and can be attributed to polygenic influences and environmental factors [3]. Epidemiological studies show strong correlation between Alzheimer's disease and metabolic syndrome demonstrating that the lipoprotein profile (ApoE_4_ homozygous genotype), hyperinsulinemia and type II diabetes mellitus are among the most characteristic prognostic factors [3]. The secretion of CNS neurohormones, the master regulators of these endocrine and metabolic conditions is considerably altered by senescence [4]. Changes in corticotrophin releasing hormone (CRH), luteinizing hormone-releasing hormone (LHRH), and GHRH secretion related to obesity, hyperinsulinemia and altered leptin signaling may play a role in the development of Alzheimer's disease [5]. Besides, hypothalamic neurohormones have been shown to have intrinsic activity on both cognitive processes [6-10] and the development of Alzheimer's disease [6, 7, 9-11]. In the same category, exogenous GHRH has recently been demonstrated to impair hippocampal memory consolidation [12] while antagonists of LHRH and GHRH showed a positive impact on learning and memory [6, 7, 9-11]. Data in the literature are not in complete agreement regarding the etiopathogenetic role of the GHRH-GH-IGF-I system in the development of senile dementia [13-17].

Since centrally released GHRH controls the whole GHRH-GH-IGF-I axis, a novel and very potent antagonist of GHRH (designated MIA-690), synthesized in our laboratory, was tested *in vivo* and *in vitro* in different models of Alzheimer's disease to clarify the effects of GHRH on the progress of symptoms and to elucidate the mechanism of action. Transgenic mice (5XFAD strain), that develop neurodegenerative symptoms characteristic of Alzheimer's disease were used *in vivo*. These genetically engineered mice accumulate amyloid-β_1-42_ plaques as a result of presenilin-1 (PSEN-1) and APP mutations. Proper cleavage is hindered due to both substrate and enzyme mutations [18]. In behavioral studies, spatial learning and memory of the transgenic mice treated with the GHRH antagonistic analog were recorded and followed up with the help of Morris water maze (MWM), for 6 months. The studies, *in vitro*, tested the effects of the analog on human neural degeneration evoked by amyloid-β_1-42_. Human cortical neuronal stem cells (HCN-2) were cultured, differentiated and subjected to co-treatment with amyloid-β_1-42_ and our GHRH antagonist. The viability of the cells was measured by proliferation assays, while changes in gene expression in autopsied brain samples were detected by “Mouse Alzheimer's Disease” real-time RT-PCR Array. The alterations of nucleic acid metabolism were later confirmed by analysis of protein changes using enzyme-linked immunosorbent assays (ELISA). A special emphasis was placed on the proteomic detection of the expression of IGF-I, IGF-II and brain derived neurotrophic factor (BDNF). Since oxidative stress and inflammation are key pathologic processes in Alzheimer's disease [1], direct assessment of reactive oxygen species formation was also performed by aminophenyl-fluorescein (APF) assays, while the expression of superoxide dismutase 1 (SOD1) and glutathione-peroxidase 1 (GPx1) was determined by ELISA. The extent of the accumulation of amyloid-β and total τ was determined by ELISAs from the necropsied brain samples of 5XFAD mice.

## RESULTS

### Morris water maze experiments

**Figure 1:**
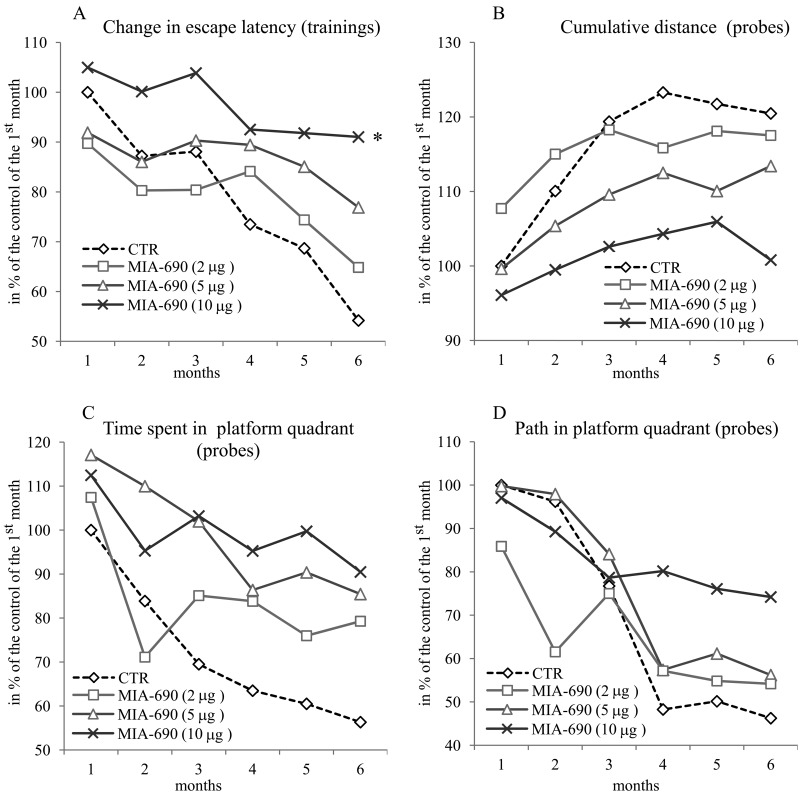
The effect of the GHRH antagonist, MIA-690, on the progressive changes of the behavioral parameters of the 5XFAD transgenic mice in Morris water maze (MWM) experiments. Mice were treated with daily subcutaneous injections of GHRH antagonist MIA-690 at doses of 2, 5, and 10 μg for 6 months. The pooled standard errors (PSE)s of the groups were the following (**A**) control: 36.0, MIA-690 (2 μg): 41.2, MIA-690 (5 μg): 33.3, MIA-690 (10 μg): 36.9; (**B**) control: 21.8, MIA-690 (2 μg): 31.4, MIA-690 (5 μg): 20.6, MIA-690 (10 μg): 16.0; (**C**) control: 53.1, MIA-690 (2 μg): 63.4, MIA-690 (5 μg): 51.9, MIA-690 (10 μg): 38.0; (**D**) control: 43.6, MIA-690 (2 μg): 74.6, MIA-690 (5 μg): 45.3, MIA-690 (10 μg): 40.0. * = p < 0.05 vs. control according to repeated measure general linear model analysis.

**Figure 2:**
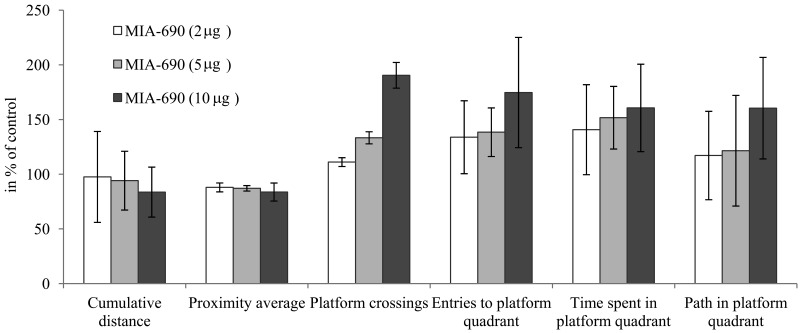
The effect of MIA-690 on the progressive changes of the probe parameters of the 5XFAD transgenic mice in Morris water maze (MWM) experiments. Mice were treated with daily subcutaneous injections of GHRH antagonist MIA-690 at doses of 2, 5, and 10 μg for 6 months. Data are represented as mean ± SEM.

**Figure 3:**
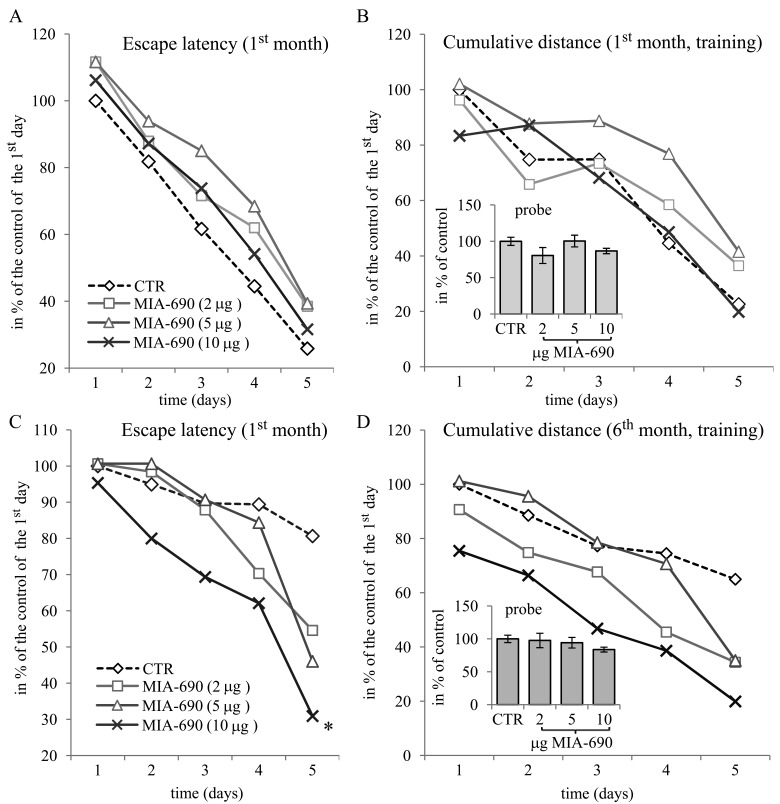
The effect of MIA-690 on the behavioral parameters of the 5XFAD transgenic mice in the spatial acquisition of the MWM experiments, during the 1^st^ and 6^th^ month. Mice were treated with daily subcutaneous injections of GHRH antagonist MIA-690 at doses of 2, 5, and 10 μg for 6 months. The pooled standard errors (PSE)s of the groups were the following (**A**) control: 28.32, MIA-690 (2 μg): 44.1, MIA-690 (5 μg): 25.5, MIA-690 (10 μg): 37.6; (**B**) control: 30.8, MIA-690 (2 μg): 47.4, MIA-690 (5 μg): 25.0, MIA-690 (10 μg): 43.2; (**C**) control: 20.1, MIA-690 (2 μg): 15.9, MIA-690 (5 μg): 17.6, MIA-690 (10 μg): 28.6; (**D**) control: 22.0, MIA-690 (2 μg): 16.6, MIA-690 (5 μg): 19.8, MIA-690 (10 μg): 23.5. * = p < 0.05 vs. control according to repeated measure general linear model analysis.

**Figure 4:**
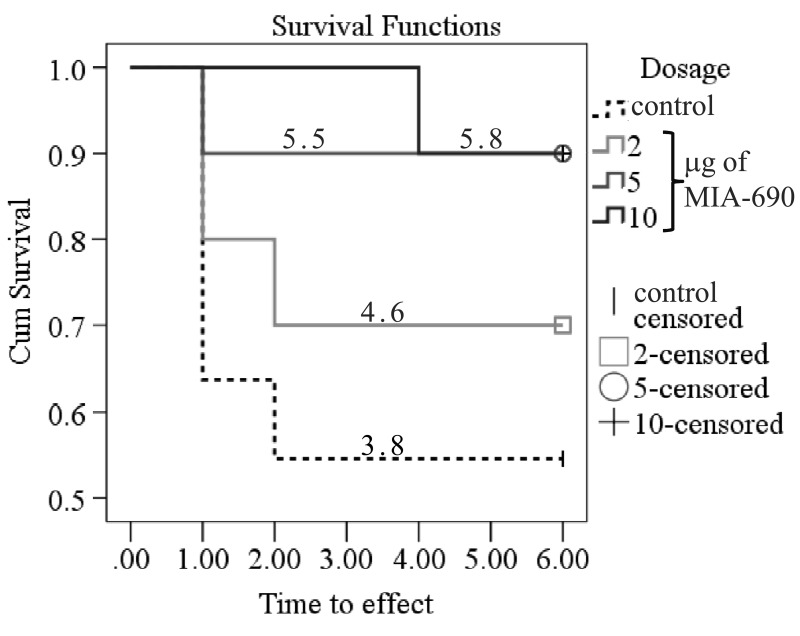
The effect of MIA-690 on the survival of the 5XFAD transgenic mice over 6 months. Numbers on each line represent the estimated mean survival time for each group. Mice were treated with daily subcutaneous injections of GHRH antagonist MIA-690 at doses of 2, 5, and 10 μg for 6 months.

**Figure 5:**
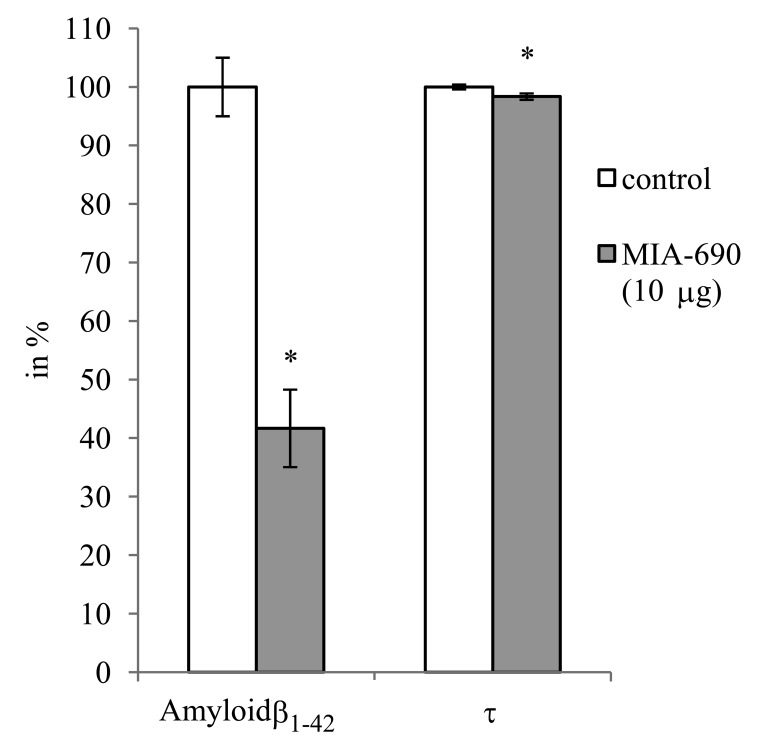
The effect of MIA-690 on the accumulation of amyloid-β_1-42_ and τ-protein in the brain of 5XFAD transgenic mice. Mice were treated with daily subcutaneous injections of GHRH antagonist MIA-690 at doses of 2, 5, and 10 μg for 6 months. * = p − 0.05 vs. control. Data are represented as mean +/− SEM

During the 6 month observation period, a marked deterioration of spatial learning was observed according to the probe parameters and the decrease in latency seen during the acquisition phase (Fig. 1 A, B, C, D). Repeated measure of general linear model (GLM) revealed that 10 μg MIA-690 almost completely abolished the progressive decrease in the amplitude of the latency curve (Fig. 1 A; Between-Subject F_3,37_=831.73, p<0.01, Tukey's *post hoc* test: p<0.05 vs. control). The analog also showed a tendency to attenuate the changes in other behavioral parameters (Fig. 1 B, C, D and 2). After six months, conspicuous differences could be observed between the control and the MIA-690 groups during the acquisition period, especially when compared to the results of the first month (Fig. 3 A, B, C, D). The group treated with 10 μg MIA-690 performed significantly better and the effect in latency proved to be statistically significant (Fig. 1 C; Between-Subject F_3,28_=64.37, p<0.01, Fisher's *post hoc* test: p<0.05 vs. control). Further, the analog appeared to prolong survival, although this effect did not prove to be statistically significant (Fig. 4). The analysis of the necropsied brain samples demonstrated that the effective concentration (10 μg) of the GHRH antagonist dramatically decreased the cerebral deposition of amyloid-β_1-42_ (MIA-690 *t*_(78)_=7.025 and p<0.05 vs. control) and slightly attenuated the total accumulation of τ-protein (MIA-690 *t*_(78)_=2.395 and p<0.01 vs. control) in the transgenic mice (Fig. 5).

### HCN2 human cortical cell cultures

**Figure 6:**
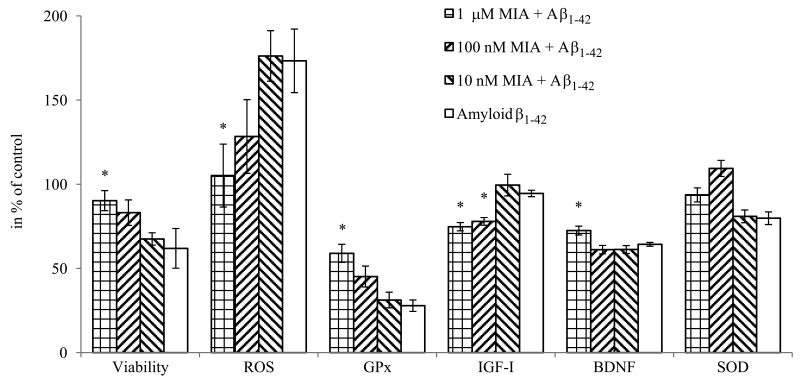
The effect of MIA-690, on the viability, free radical formation, enzyme and mediator expression of HCN-2 cells in vitro. Cells were treated with 10 μM amyloid-β_1-42_, and the combination treatments with 10 μM amyloid-β_1-42_ and the 3 doses (10 nM, 100 nM and 1 μM) of MIA-690. Abbreviations: ROS: reactive oxygen species, GPx: glutathione-peroxidase, BDNF: brain derived neurotrophic factor. * = p < 0.05 vs. control. Data are represented as mean +/− SEM.

MIA-690 exerted dose-dependent effects on the viability, oxidative metabolism and mediator release of HCN-2 cells (Fig. 6). The peptide remarkably attenuated the toxic impact of co-treatment with amyloid-β_1-42_ on the viability of neurons (F_4,23_=2.8, p<0.05, Fisher's *post hoc* test: p<0.05, 10 μM MIA-690 + amyloid-β_1-42_ vs. amyloid-β_1-42_) and practically abolished the generation of ROS evoked by amyloid-β_1-42_ co-treatment (F_4,43_=2.64, p<0.05, Fisher's *post hoc* test: p<0.05, 1 μM MIA-690 + amyloid-β_1-42_ vs. amyloid-β_1-42_). While the analog did not have a significant and linear impact on SOD1 expression it significantly increased the glutathione-peroxidase (GPx) (F_4,75_=15.2, p<0.01, Tukey's *post hoc* test: p<0.05, 1μM MIA-690 + amyloid-β_1-42_ vs. amyloid-β_1-42_) and brain derived neurotrophic factor (BDNF) (F_4,75_=58.72, p<0.01, Fisher's *post hoc* test: p<0.01, 1 μM MIA-690 + amyloid-β_1-42_ vs. amyloid-β_1-42_) expression at the highest applied concentration. The GHRH analog also suppressed the release of IGF-I (F_4,75_=9.22, p<0.01), (Tukey's *post hoc* test: p<0.05, 100 nM MIA-690 + amyloid-β_1-42_ vs. amyloid-β_1-42_ and p<0.01, 1 μM MIA-690 + amyloid-β_1-42_ vs. amyloid-β_1-42_), but its effect on the secretion of IGF-II was negligible (data not presented). The PCR Array studies revealed statistically significant changes in the expression of 22 Alzheimer's disease related genes in the brain samples of the 5XFAD mice following treatment with 10 μg MIA-690 for six months (Table 1).

**Table 1:** Expression of genes related to Alzheimer's disease in the brain samples of 5XFAD transgenic mice treated with 10 μg MIA-690 daily for 6 months

Gene		Fold change vs. controls
*Acetylcholinesterase*		-1.61
*Amyloid-β (A4) precursor protein binding*	*family A, member 2*	-2.94
	*family B, member 3*	-2.04
*Amyloid-β (A4)*	*precursor protein*	-1.79
	*precursor-like protein 2*	-1.59
*Anterior pharynx defective 1a homolog*		-1.54
*β–site APP-cleaving enzyme 2 (BACE2)*		-2.27
*Caspase 4, apoptosis-related cysteine peptidase*		-3.23
*Cyclin-dependent kinase 1*		-2.00
*Clusterin*		-1.72
*Cathepsin*	*C*	-6.67
	*G*	-2.22
*Growth associated protein 43*		-1.61
*Insulin-like growth factor 2*		-3.22
*Low density lipoprotein receptor-related protein 6*		-4.76
*Microtubule-associated protein*	*τ*	-1.33
	*2*	-1.54
*Neural precursor cell expressed and developmentally down regulated gene (NEDD)8 activating enzyme E1 subunit 1*		-4.35
*Nicastrin*		-1.69
*Presenilin*	*1*	-2.17
	*2*	-1.27
*Ubiquinol cytochrome c reductase core protein 2*		1.49

The expression of multiple genes related to Alzheimer's disease was evaluated using real-time RT2 Profiler PCR Array system. The table lists the genes of interest evaluated and their fold increase or decrease in brains obtained from mice treated for 6 months with MIA-690. Data represent fold differences of individual gene expression between MIA-690 treated animals vs. controls. Positive values indicate up-regulation of individual genes; negative values indicate down-regulation. Five experiments were run for each study group. The data were evaluated by two-tailed Student's t test. Only genes with statistically significant changes (p < 0.05) are represented.

## DISCUSSION

Our previous studies clearly demonstrated that the GHRH antagonists developed by us [19, 20] had a strong impact on the GHRH - GH – IGF-I axis [21]. The effects of GHRH antagonists on several other physiologic and pathologic conditions have already been demonstrated [19, 22-28]. The role of the GHRH - GH – IGF-I axis in the regulation of learning processes and the development of Alzheimer's disease, however, has not been completely clarified. Both hyperactivity [29, 30] and hypoactivity [31] of the system have been connected to decreased life-expectancy and impaired cognition. Nevertheless, important recent observations support the view that the suppression of the axis can improve cognition in both physiologic and pathologic conditions [7, 10, 11, 15, 17].

Present experiments demonstrate that the GHRH antagonist, MIA-690, has several beneficial effects in each of the different models of Alzheimer's disease. Regarding the GHRH-GH-IGF axis, MIA-690 decreased the secretion of IGF-I (Fig. 6) in the supernatant of HCN-2 cell cultures which is critical since in Alzheimer's disease one of the most important pathologic phenomena, is the competition of insulin and amyloid-β for insulin-degrading enzyme (IDE). In hyperinsulinemic states, insulin, which has higher affinity to IDE, occupies the binding sites of the enzyme, rendering IDE inaccessible to amyloid-β, hence amyloid-β accumulates [17]. In addition to insulin, IGF-I, which is the most abundant member of the IGF family in the CNS, appears to influence cognitive decline in pathologic conditions [5, 13, 16, 17]. Decreased IGF-I signaling appears to ameliorate, directly, the amyloid-β proteo-toxicity by enhancing DNA repair and increasing resistance to oxidative stress [14, 15].

In the *in vivo* studies, the GHRH antagonist significantly and dose-dependently delayed the Alzheimer's disease-related deterioration of the acquisition phase in MWM (Fig. 1A, Fig. 3C). The peptide also tended to improve the parameters of cognitive performance by the 6^th^ month of the follow-up period as reflected by the probe values (especially the cumulative distance and platform crossings) of spatial reference memory (Fig. 1B, C, D, Fig. 2, Fig. 3D). The PCR Array studies (Table 1), revealed that the neuro-peptide analog, beside several possible, long-term activities, may have acute beneficial effects on learning. This is in harmony with our previous findings [7], and confirms the inhibitory activity of intranasal GHRH agonists on hippocampal memory formation [12]. MIA-690 increased the expression of ubiquinol-cytochrome c reductase core protein 2, which suggests that the GHRH antagonist may restore impaired cellular respiration [32]. In contrast, MIA-690 decreased the expression of acetylcholinesterase, which is consequential, considering the key role of acetylcholine in hippocampal learning [33]. Further, the inhibition of acetyl-cholinesterase is one of the most important, currently available, palliative treatment options for Alzheimer's disease [34].

Our previous publications have already demonstrated that different classes of hypothalamic neurohormone analogs could influence CNS functions. For example, the LHRH antagonist, cetrorelix, facilitated memory and had anxiolytic and antidepressive actions in mice [6] and rats [9] exposed to the neurotoxic effects of an amyloid-β fragment (amyloid-β_25-35_). In a similar fashion, the GHRH antagonist, MZ-4-71, improved memory consolidation in passive avoidance learning [7, 10], decreased anxiety [7, 35], and proved to be antidepressive [7, 36], in CFLP mice treated with amyloid-β_25-35_. In a different model of cognitive decline, the treatment of senescence accelerated mice (SAMP8) with another GHRH antagonist (MZ-5-156) also enhanced cognitive functions [11]. The GHRH antagonist treatment produced significantly better performance in active avoidance T-maze, step-down passive avoidance and object recognition. Also, the peptide improved pole balance and increased survival and telomerase activity. These findings are in complete agreement with our studies, as MIA-690 treatment also showed a tendency to prolong survival (Fig. 4).

Our genomic and proteomic studies shed light on further possible mechanisms of action of MIA-690. The GHRH antagonist influenced the transcription of almost 2 dozen putative Alzheimer's disease markers according to the PCR Array experiments (Table 1). The most notable examples are related to the metabolism of amyloid-β, the microtubule system, apoptosis, neural signal transduction, and energy homeostasis. Regarding the metabolism of amyloid-b, the transcriptional studies revealed a remarkable inhibition of the expression of *APPs* and the *amyloid-β precursor protein-binding proteins (APP-BP)s*. Further, the GHRH antagonist decreased the transcription of the amyloid-β generating *BACE2* and several components (*presenilin 1, presenilin 2, anterior pharynx-defective 1 and nicastrin*) of the γ-secretase complex [37]. The pathogenic role of cathepsins in the development of Alzheimer's disease needs further clarification [38] since they can play a role in both the generation of and the degradation of amyloid peptides. In our experiments, substantial decreases could be detected in the “C” and “G” members of the family. The observed down-regulation of *low density lipoprotein receptor-related protein (LRP)6* provide further evidence that MIA-690 can influence amyloid-β degradation, since LRPs, in cooperation with α2-macroglobulin and ApoE, are the main factors in the modulation of amyloid-β secretion/breakdown [1, 39].

Similar changes are observed in the expression of the *microtubule-associated proteins (MAP)s*. Both *MAP2* and *MAPτ* were down-regulated. The final markers of these genomic changes (amyloid-β_1-42_ and total τ levels) were verified by proteomic determination. The amyloid-β_1-42_ level showed especially dramatic decrease due to MIA-690 treatment (Fig. 5), which finding suggests that further studies are needed and should include the selective measurement of hyperphosphorylated as opposed to total τ levels. This also confirms that the relationship between amyloid-β_1-42_ raft formation and τ-based neurofibrillary tangle formation is not direct but is a highly unpredictable and cross-related process of the deranged axonal transport [40]. The importance of hyperphosphorylation is accentuated by the marked down-regulation of *cyclin-dependent kinase 1 (CDK)1*. CDKs belong to those “culprits”, which take part in the post-translational modifications of the MAPs [41], and catalyze, with phosphorylation, the misfolding of τ fibrils.

An important feature of Alzheimer's disease is the connection of the cerebral amyloid process to neural regeneration via the γ-secretase complex. As a double-edged sword, γ-secretase is the rate limiting enzyme of not only amyloid-β production, but also Notch signaling. This, in turn, regulates cell renewal in the CNS [42]. Therefore, overburdening of γ-secretase by amyloid-b accumulation, not only accelerates apoptosis and neuronal degeneration but may also decrease the neurogenesis mediated by Notch signaling [43]. The decrease in *APP* and *BACE2* expression and amyloid-β_1-42_ production evoked by MIA-690 may preserve the regenerative potential of the CNS. This concept is supported by the down-regulation of *growth associated protein 43*, which seems to play a pathologic role in the abnormal hyper-activation of hippocampal cells and the dysfunctional signal transduction seen in Alzheimer's disease [44]. Regarding regenerative processes, however, the most important finding is that treatment with MIA-690 significantly increased the secretion of BDNF (Fig. 6); this protective neurotrophin is a well-established and adversely affected marker of neurodegenerative processes [1, 45].

The attenuation of oxidative stress by GHRH antagonists [11] or by the decrease in IGF-I signaling [15] also seems to play a crucial role in the protection against amyloid-β proteotoxicity. In our experiments, MIA-690 significantly decreased free radical formation (Fig. 6) of HCN-2 cells. Further, this GHRH antagonist dose-dependently augmented GPx1 levels while it did not have a significant effect on SOD1 secretion (Fig. 6). Although, Banks *et al.* found a slight decrease in GPx expression, this by no means indicates a contradiction. It appears that MIA-690, in sharp contrast to the earlier GHRH antagonist that he used, directly stimulates the protective antioxidant enzymes in the CNS. Therefore, in contrast to our previous experiments, present findings demonstrate that the activity of the anti-oxidative system in the MIA-690 treated animals is not only passively responsive to the free radical burden but actively up-regulates the relevant ROS catabolic enzymes. The oxidative stress elicited by proteo-toxicity inevitably leads to apoptosis [1, 15]. In full agreement with our anti-oxidative experiments, MIA-690 dose-dependently increased the viability of HCN-2 cells treated by amyloid-β_1-42_ (Fig. 6). The GHRH antagonist also attenuated the transcription of *caspase* and *clusterin* (Table 1.), both of which play important roles in apoptotic processes in the CNS [1, 46]. Further, GHRH antagonist treatment decreased the expression of *APP-BPs* and the *neural precursor cell-expressed and developmentally down-regulated gene (NEDD)8 activating enzyme E1-subunit 1*, which both cooperate in neddylation, one of the post-translational tagging processes that can lead to apoptosis [47].

To properly analyze present results and harmonize them with literature data, it is necessary to differentiate between two effects and mechanisms. Inhibition of the different levels of the GHRH-GH-IGF axis apparently exerts a beneficial impact on the progress of Alzheimer's disease [7, 10, 11, 15, 17]. This phenomenon, however, should not be confused with the effect of these mediators on cognitive performance, *per se*. Concerning Alzheimer's disease, literature data suggest that the inhibition of the axis can be desirable, at least, in the developing disorder. [7, 10, 11, 14-17]. However, regarding the acute CNS effects, the available findings are more ambiguous, and raise the possibility, that, in cognitive tests, an adverse, acute effect on learning can mitigate the consequences of the activities on neuro-degeneration. Accordingly, while GHRH antagonists enhance [7, 10] and exogenous GHRH impairs [12] memory consolidation, IGF-II appears an important acute stimulator of this process [48]. Although, IGF-II is far less dependent on the GHRH-GH axis than IGF-I [49], it is important to emphasize that in our experiments, chronic peptide administration decreased IGF-II expression in the brain samples (Table 1.). Therefore, albeit acute administration of MIA-690 did not influence IGF-II secretion in HCN-2 tissue cultures (data not published), the direct effect of the peptide on memory can interfere with the beneficial actions on neurodegeneration. This hypothesis is strongly supported by the recent findings of Vitiello *et al.* [50] and Baker *et al.* [51]. It seems that the activation of the GHRH-GH-IGF-I axis has rejuvenating action on the cognitive performance of the elderly, similar to its somatic effects [52]. Recently it was shown that the mammalian target of Rapamycin (mTOR) pathway is involved in cellular senescence, organ aging and diseases of aging including Alzheimer's disease [53]. The mTOR pathway was also implicated as a molecular link between growth and aging [54]. The GHRH-GH-IGF-I axis may concurrently pathologically activate mTOR [53] and the cascade mechanisms involved in oxidative, inflam-matory, degenerative and neoplastic processes [1, 11, 14, 15, 17, 19]. Interestingly, while a reduction of insulin/IGF-I signaling can result in diabetes, its reduction can also increase longevity and delay the onset of protein-aggregation-mediated toxicity [14]. This phenomenon, called the “insulin paradox”, can be explained by mTOR mechanisms: if low insulin/IGF-I signaling does not activate mTOR, then it is beneficial for longevity and health [55]. However, if insulin/IGF-I signaling is low, because of the blockage caused by the feedback of the over-activated mTOR, then it is harmful [55].

Taken together, our data suggest that further studies are needed to clarify the mechanism of action of GHRH analogs in models of Alzheimer's disease, with special emphasis on the separation between the acute effects on memory formation and the selective impact on proteo-toxicity itself. The direct and indirect, short-term and long-term activities should also be further elucidated, taking into consideration the bewilderingly complex nature of the GHRH-GH-IGF axis. Our compounds freely penetrate the blood-brain barrier [56] and apparently target different levels of the pathologic cascade of Alzheimer's disease inhibiting aggregation and proteo-toxicity while restoring normal neural metabolism and regeneration. In making the choice of which GHRH antagonist to use, focusing on those properties relating to the CNS undoubtedly will have important clinical therapeutic application.

## METHODS

### Ethics Statement

Investigation has been conducted in accordance with ethical standards, according to the Declaration of Helsinki, and in accord with national and international guidelines and has been approved by the authors' institutional review board.

### Peptides

The GHRH antagonist peptide, MIA-690, was synthesized in our laboratory by the solid-phase method and purified by reversed-phase HPLC as described previously [20]. The structure of MIA-690 is: [(PhAc-Ada)^0^-Tyr^1^, D-Arg^2^, Cpa^6^, Ala^8^, Har^9^, Fpa_5_^10^, His^11^, Orn^12^, Abu^15^, His^20^, Orn^21^, Nle^27^, D-Arg^28^, Har^29^] hGH-RH_[1-29]_NH2]. Noncoded amino acids and acyl groups are abbreviated as follows: Abu, α-aminobutyric acid; Ada, 12-aminododecanoyl; Cpa, parachlorophenylalanine; D-Arg, D-arginine; Har, homoarginine; Fpa_5_, pentafluoro phenylalanine; hGHRH, human GHRH; Nle, norleucine; Orn, ornithine; PhAc, phenylacetyl. For treatment, MIA-690 was dissolved in an aqueous solution of 0.1% DMSO (Sigma) and 10% propylene glycol (Sigma-Aldrich, St. Louis, MO).

### Behavioral studies

Transgenic mice (5XFAD strain) were obtained from The Jackson Laboratories (Bar Harbor, ME). The animals were housed in sterile cages in a temperature-controlled room with a 12-h light/12-h dark schedule and were fed with autoclaved chow and water, *ad libitum*. Both sexes were used, evenly distributed between the different treatment groups. For the Morris water maze experiments 41 adult mice (approximately 3 months old) were used and were divided into 4 treatment groups, each of which received the following subcutaneous daily treatment for 6 months: group 1: (control), vehicle solution; group 2: MIA-690 (2 μg); group 3: MIA-690 (5 μg); and group 4: MIA-690 (10 μg).

The maze is a circular white steel pool (120 cm diameter; 40 cm high). The pool was filled to approximately 30 cm with water at room temperature (22 ± 2 °C). The water was made opaque with non-toxic white liquid tempura paint (Crayola, Easton, PA). The black-furred animals provided sufficient contrast for video tracking. The experiment was performed according to the guidelines of the literature [57-59]. The pool was divided virtually into four quadrants with four equidistant release points around the edge. The release points were labeled according to the points of compass: south (S), west (W), north (N), east (E) [59]. The goal platform (10 cm diameter) was submerged 1.5 cm beneath the water surface in the center of one of the quadrants (at 30 cm radial distance from the rim of the pool). The platform positions were labeled according to the nomenclature of the recording software (Water Maze Software, Columbus Instruments, Columbus, OH) and the compass directions: 1, north-west (NW); 2, south-west (SW); 3, south-east (SE); 4, north-east (NE) [59]. For each training trial, a mouse was released at a semi-randomly assigned release point and allowed to swim freely. Once the platform was reached, the mouse was allowed to remain there for 15 s. If the platform was not located after 60 s, the mouse was gently guided to the platform and allowed to remain there for 15 s. Trials of individuals were separated by about 70-80 min (one trial lasted for almost 2 min) and the mice were dried between trials to prevent hypothermia. Differences in swimming speed, motivation and tendency to float were first assessed during 2 pre-liminary sessions, when the platform was visible; and “floaters” were excluded from the study. During a daily session, each animal then received a block of 4 training trials with the platform hidden; one complete training cycle consisted of 5 consecutive days. The platform remained in the same quadrant for all acquisition trials and for all mice. At the end of the training, on the 6^th^ day, a probe test was completed wherein the platform was removed from the pool and each mouse was allowed to search freely for 60 s. A video-tracking system was used to monitor and quantify performance (Videomex-One hardware and Water Maze Software, Columbus Instruments, Columbus, OH). For all trials, peripheral cues around the maze environs remained constant throughout testing. The observed and recorded parameters for the trainings were: escape latency, path length, cumulative distance (CD) and proximity average (PA), while for probes CD, PA, platform crossings (PC), entries to platform quadrant (EPQ), path length in platform quadrant (PPQ) and time spent in platform quadrant (TPQ) were used. Averages of the output variables of the four individual trials were used for comparison and statistical evaluation. Mice were followed monthly for 6 months (between the age of 3 and 9 months) after commencing their treatment. In addition to the survival of mice, their training and probe values were recorded monthly. For comparison of the probe values, the CD, the PC, the EPQ, the PPQ and the TPQ were used but the change in escape latency between the first and the fifth day was also found to be a sensitive marker. Between the monthly sessions the probe sessions facilitated extinction of memory and for the next training session the platform was semi-randomly relocated to a new position.

At the end of the experiment, the mice were sacrificed by cervical dislocation and decapitation, necropsy was performed, and the brains were removed. The hemispheria were immediately snap-frozen in liquid nitrogen and stored at −80 °C for PCR and proteomic studies. For the determination ofamyloid-β_1-42_ and total τ-protein levels, mouse-specific ELISA kits were used according to the manufacturer's instructions (Invitrogen, Carlsbad, CA).

#### *In vitro* studies

### Cell cultures

Investigations, *in vitro*, followed, in general the descriptions in the literature, with slight modifications [60]. Briefly, HCN-2 cells (American Type Culture Collection, Manassas, Virginia, USA) were cultured in DMEM medium (supplemented with 10% Fetal Bovine Serum (FBS) and 0.1% penicillin/streptomycin) at 37°C and in an atmosphere of air and 5% CO_2_. At 70-80 percent of confluency, the cultures were trypsinized and resuspended in fresh serum-containing medium and either transferred into T-75 flasks or directly plated into 48- well micro plates at 10,000 cells/cm^2^. The next day, the differentiation of HCN-2 cells was induced by adding fresh medium containing 25 ng/ml NGF, 0.5 mM dibutyryl cAMP and 0.1 mM isobutylmethylxanthine (IBMX) (all from Sigma-Aldrich, St. Louis, MO) for a week. Then, human amyloid-β_1-42_ (Abbiotec LLc, San Diego, CA)stock solution (10 mM) was prepared in DMSO and then immediately diluted to appropriate concentrations in the assay medium. The medium used for neurotoxicity assay was N2-supplemented DMEM/F12 (Gibco BRL, NY) with 10% FBS. The treatment groups were control, amyloid-β_1-42_, and the combination treatments with amyloid-β_1-42_ and the 3 doses (10 nM, 100 nM and 1 μM) of MIA-690; controls received propylene glycol and DMSO containing medium. The effect of the analog on proteo-toxicity was evaluated after three days of exposure. The viability of the cells was determined by using the 3-(4,5-Dimethylthiazol-2-yl)-2,5-diphenyltetrazolium bromide (MTT) assay (Cell Titer 96® Non-Radioactive Cell Proliferation Assay, Promega, Madison, WI), according to the manufacturer's instructions [61]. APF assay (Invitrogen) was used for the detection of free radical formation according to the manufacturer's instructions. Concentrations of the specific proteins (IGF-I, IGF-II, GPx1, SOD1, BDNF) in the media were determined using appropriate ELISA kits according to the manufacturer's instructions. IGF-I, IGF-II and BDNF human ELISA kits were obtained from AbCam Inc. (Cambridge, MA) while GPx1 and SOD1 kits from Abfrontier through Biovendor, LLC, (Candler, NC). Readings were normalized to protein concentrations as determined by NanoDrop (NanoDrop Technologies Inc., Wilmington, DE).

### Total RNA isolation, reverse transcription real-time PCR array

Total RNA was isolated and DNAse treated from representative hemispheria using NucleoSpin kit according to the manufacturer's instructions (Macherey-Nagel Inc., Bethlehem, PA). Five samples each from the control and the 10 μg MIA-690 group were analyzed. The yield and the quality of RNA samples were determined spectrophotometrically using 260 nm, and 260/280 and 260/230 nm ratio. The synthesis of cDNA was performed as described [62]. Briefly, 1 μg of RNA from each sample was reverse-transcribed into cDNA by RT First Strand kit (Qiagen). Reverse transcription was done in a Veriti 96-well thermal cycler (Applied Biosystems). The Mouse Alzheimer's Disease real-time quantitative PCR array (PAMM-057Z Qiagen) used in our study contains 84 unique genes related to Alzheimer's disease. All PCR arrays were performed using iQ5 Multicolor Real-Time Detections System (Bio-Rad). All genes represented by the array showed a single peak on the melting curve characteristic of the specific products. Data analysis of gene expression was performed using Excel based PCR Array Data Analysis Software provided by the manufacturer (Qiagen): fold-changes in gene expression were calculated using the ΔΔCt method and five stably expressed housekeeping genes (Actb, B2m, Gapdh, Gusb, Hsp90ab1) were used for normalization of the results.

### Statistical analyses

Statistical evaluation of the *in vivo* experiments was performed by repeated measure General Linear Model (GLM) or survival analysis (Kaplan-Meier). GLM was followed by Tukey's and Fisher's *post hoc* tests, while for the survival analysis the Log Rank (Mantel-Cox) test was used for group-wise comparisons. The *in vitro* data were evaluated using *t*-test for independent samples or univariate GLM; the latter being followed by *post hoc* comparison. Results are expressed either as the means ± SEM or as means and pooled standard errors (PSE)s, in the case of line plots. Differences with p<0.05, compared to the control, were considered statistically significant. Data reductions and statistical analyses were performed by SigmaPlot 12.0 (Systat Software, Inc., Chicago, IL) and IBM SPSS Statistics 20.0 (IBM Corporation, Armonk, NY).
